# Case Report: The Formation of a Truncated PAX5 Transcript in a Case of Ph-Positive Mixed Phenotype Acute Leukemia With dic(7;9)(p11-p13;p13)

**DOI:** 10.3389/fonc.2021.703612

**Published:** 2021-08-26

**Authors:** Yan Yu, Zhao Zeng, Jundan Xie, Qiongyu Lu, Wenzhi Cai, Ruixi Zhang, Jinlan Pan, Yun Zhao, Aining Sun, Huiying Qiu, Suning Chen

**Affiliations:** ^1^Jiangsu Institute of Hematology, National Clinical Research Center for Hematologic Diseases, The First Affiliated Hospital of Soochow University, Medical College of Soochow University, Suzhou, China; ^2^Collaborative Innovation Center of Hematology, Soochow University, Suzhou, China; ^3^Institute of Blood and Marrow Transplantation, Soochow University, Suzhou, China; ^4^Cyrus Tang Hematology Center, Soochow University, Suzhou, China; ^5^Key Laboratory of Thrombosis and Hemostasis of Ministry of Health, Suzhou, China

**Keywords:** Pax5, UBE2D4, MPAL, BCR/ABL, dic(7;9)

## Abstract

PAX5 plays a critical role in B-cell precursor development and is involved in various chromosomal translocations that involve the fusion of a portion of PAX5 to at least 49 different partners reported to date. Here, we identified a novel PAX5 fusion transcript in a Ph-positive mixed phenotype acute leukemia case with dic(7;9)(q13;q13), in which a translocation juxtaposes the 5’ region of PAX5 and the ubiquitin-conjugating enzyme E2D4 (UBE2D4) to generate a PAX5-UBE2D4 fusion gene. To further explore the general characteristics and function of PAX5-UBE2D4, we cloned the full-length cDNA, which was amplified from the bone marrow of the patient. Interestingly, the fusion was located in the nucleus and negatively affected PAX5 transcription activity. Importantly, the fusion promoted tumor growth in nude mice and the proliferation of NIH3T3 cells *in vitro*. In conclusion, the fusion resulted in partial oncogenic activity, in contrast to the tumor suppressor activity of wild-type PAX5.

## Introduction

The transcription factor PAX5 plays a critical role in B-cell development and differentiation and has been considered to function as a tumor suppressor in B cell precursor acute lymphoblastic leukemia (BCP-ALL). PAX5 alterations, including deletions, mutations, and rearrangements, occur in approximately 30% of BCP-ALL cases. Chromosomal rearrangements account for 2–3% of cases (1–3). It has been well reported that a number of PAX5 rearrangements give rise to in-frame fusion transcripts that encode chimeric proteins that consistently retain the PAX5 DNA binding domain at the N terminus, but the C-terminal regions are derived from various partners, including transcription factors, kinases and structural proteins (4–8). To date, at least 58 fusions have been identified, and most of them have been found in association with BCP-ALL (9). Only a limited number of the reported fusions were recurrent, such as PAX5-ETV6, PAX5-ELN, and PAX5-PML, while most have been found in single cases, such as PAX5/ASXL1 and PAX5/FOXP1 (9). In addition, half of the rearrangements have resulted in PAX5 fusions to genes in the opposite orientation, out-of-frame fusions or the expression of truncated isoforms (6). Here, we first identified a novel chromosomal dic(7;9) (p13;p13) translocation in a Ph-positive mixed phenotype acute leukemia (MPAL) patient, resulting in a PAX5 out-of-frame fusion with the ubiquitin-conjugating enzyme E2D4 (UBE2D4), which functions as a truncated PAX5. In addition, the fusion showed partial oncogenic activity, which was in contrast with the tumor suppressor ability of wild-type (WT) PAX5.

## Case Description

A 16-year-old boy was referred to our hospital in January 2010 with recurrent fever and weakness for one month. Physical examination indicated axillary lymphadenopathy and hepatosplenomegaly without anemic conjunctiva. The peripheral blood counts at diagnosis revealed multilineage cytopenia: hemoglobin 12 g/dL, white blood cells (WBCs) 12.87 x 109/L, and platelets 31 x 109/L. Bone marrow (BM) aspiration showed hypercellularity with 89.2% blasts and lymphatic changes. Flow cytometric analysis revealed that 23.4% of the BM blast cells were positive for HLA-DR, CD10, CD20, CD19, CD13, CD33, CD34, MPO and CD79a but negative for CD117, CD14, CD15, CD2, CD3, and CD7 (**Supplementary Figure 1**). Then, the patient was diagnosed with MPAL with co-expression of myeloid and B lymphoid lineage antigen according to the 2016 WHO classification. The karyotype of the bone marrow cells was 45, XY, dic(7;9)(p11-13;p13), t(9;22)(q34;q11) (8) /46, XY (9). The BCR/ABL (p190) fusion gene was detected by multiplex reverse transcription-polymerase chain reaction (RT-PCR), thereby confirming the diagnosis of Ph-positive mixed phenotype acute leukemia. The patient accepted tyrosine kinase inhibitor therapy and achieved remission, which was followed by 2 DVP chemotherapy sessions (with 70 mg daunorubicin, 4 mg vincristine and 20 mg dexamethasone). Unfortunately, the patient finally had a cytological relapse in the bone marrow and died 5 months after the initial diagnosis.

## Discussion and Conclusion

**Figure 1:**
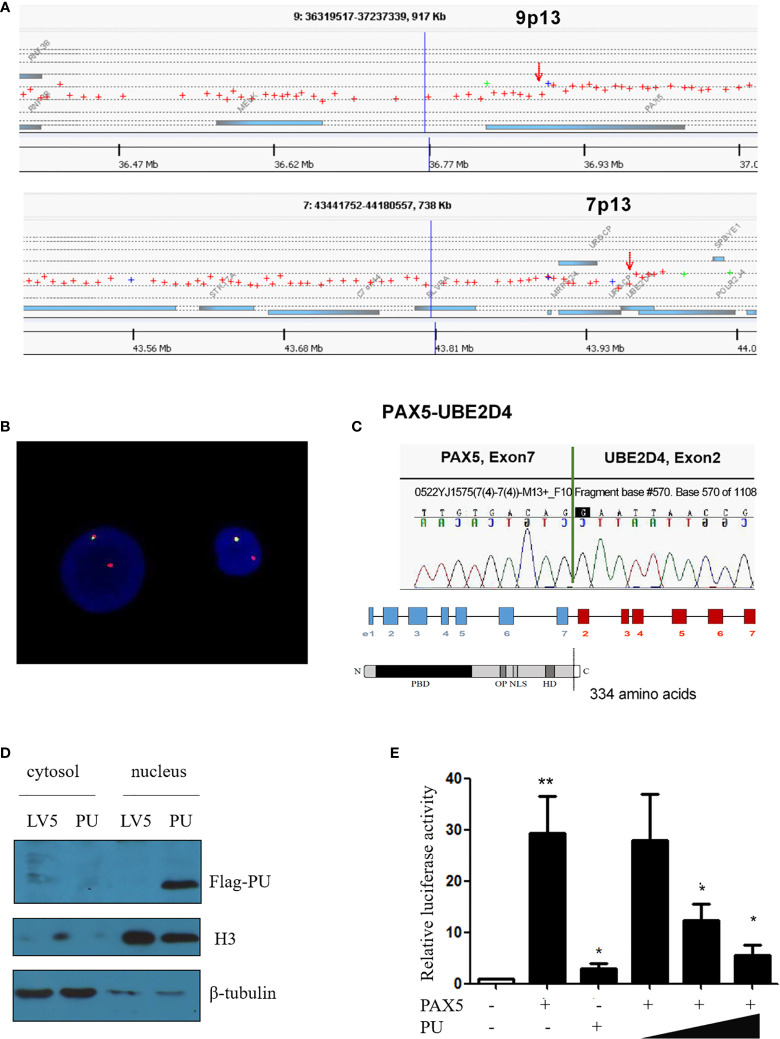
**(A)** Array comparative genomic hybridization showing the breakpoints of 9p13/PAX5 and 7p13/UBE2D4. **(B)** FISH assay showing the splitting of the N terminus (green, RP11-344B23) and C-terminus (red, RP11-652D9) of WT PAX5. **(C)** Sanger sequencing confirmed that PAX5 exon 7 was fused out-of-frame with UBE2D4 exon 2, and the schematics show the domains of the PAX5-UBE2D4 (PU) fusion protein, including PBD (DNA-binding domain), OP (octapeptide motif), NLS (nuclear localization sequence) and HD (homeodomain), and an additional 19-amino acid tail encoded by the UBE2D4 gene. **(D)** Nuclear localization of the PU fusion protein as shown by a nucleus and cytosol separation assay. **(E)** The transcription activity of the PU fusion and its dominant negative effect on PAX5 transcription activity based on the CD19 promoter luciferase reporter assay. P values are from Fisher’s exact test. *P ≤ 0.05, **P ≤ 0.01.

Based on the karyotype of the patient, array comparative genomic hybridization (array-CGH) analysis was performed, and the results indicated that the breakpoints were located in the PAX5 and UBE2D4 genes and revealed the deletion of large parts of 9p and 7p (**Figure 1A**). When using the FISH (fluorescent *in situ* hybridization) probes RP11-652D9 and RP11-344B23 corresponding to the 5’ and 3’ sequences of the PAX5 gene, respectively, we observed a red signal and a yellow signal, which was consistent with the results of the array-CGH analysis (**Figure 1B** and **Supplementary Figure 2**). Then, RT-PCR amplification revealed the presence of PAX5-UBE2D4 fusion transcripts (**Supplementary Figure 3**). Sanger sequencing confirmed the out-of-frame fusion of PAX5 exon 7 (NM_016734) with UBE2D4 exon 2 (NM_015983.4), resulting in the analogous truncated PAX5 protein with the DNA binding (PBD) domain, OCT domain and homeodomain (HD) of PAX5 and an additional 19-amino acid tail, which does not correspond to any predicted functional domain (**Figure 1C**).

**Figure 2:**
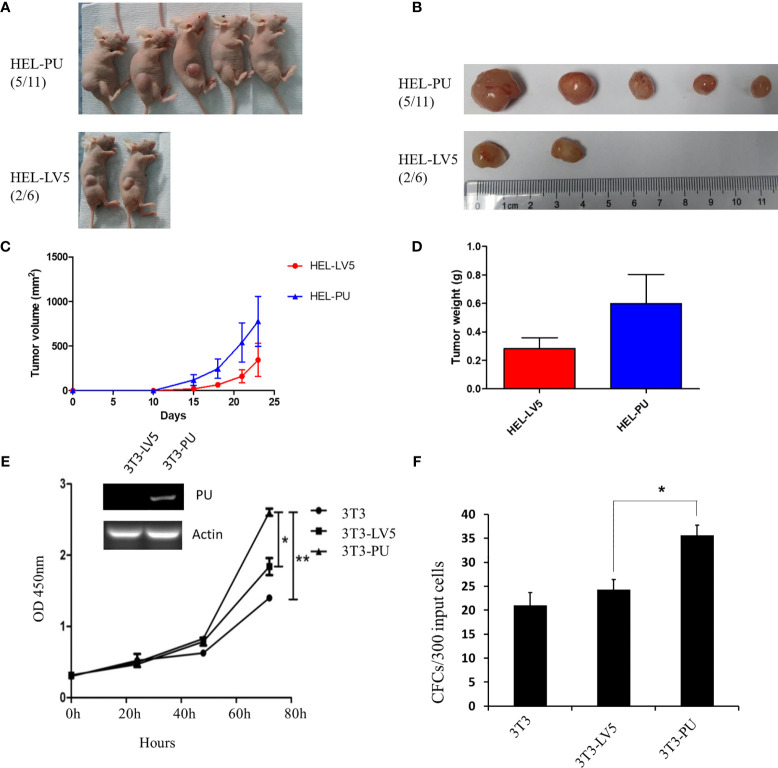
The oncogenic activity of the PAX5-UBE2D4 (PU) fusion. **(A)** PAX5-UBE2D4 (PU) increases the number of tumors generated by subcutaneous injection of HEL cells into nude mice. **(B, C)** The sizes of the tumor masses and tumor weights **(D)** after injection of HEL cells expressing the indicated genes. **(E)** PU fusion promotes the proliferation and colony formation **(F)** of NIH-3T3 cells. P values are from Fisher’s exact test. *P < 0.05, **P < 0.01.

To investigate the function of the fusion, we amplified the full-length cDNA sequence of PAX5 and UBE2D4 that was retained in the fusion found in the patient, cloned it into a lentiviral vector (LV5, GenePharm Inc., Shanghai) and the pcDNA3.1 vector, and fused it with a 3×FLAG-tag. As **Figure 1D** shows, we observed nuclear localization of the fusion, which was expected since the fusion retained the nuclear localization signal of PAX5 (**Figure 1D** and **Supplementary Figure 4**). Furthermore, we co-transfected 293T cells with the CD19 promotor-LUC construct (PGL3), pcDNA-PAX5 and increasing amounts of the pcDNA-PAX5-UBE2D4 (PU) construct. The transcription of the luciferase reporter gene was significantly downregulated in the presence of the expression of PU alone compared with that observed in the presence of wt-PAX5 (**Figure 1E**). In addition, after concomitant transfection of wt-PAX5 and PU, PAX5-driven reporter gene transcription was downregulated (**Figure 1E**), indicating the dominant-negative activity of PU. To investigate the function of PU, HEL cells were transfected with PU (HEL-PU) and the vector (HEL-LV5). Then, the cells were subcutaneously injected into 6- to 8-week-old female nude mice (n=6-11). A total of 45.5% (5/11) of mice engrafted with HEL-PU cells developed tumors, which was obviously greater than the number of mice who developed tumors in the control (HEL-LV5, 33.3%, 2/6) group (**Figures 2A, B**). The mean volume of the tumors in the PU cohort was much larger than the control cohort (**Figure 2C**). In addition, the mean weight of the tumors in the HEL-PU group was the heaviest when compared with control group (**Figure 2D**). In contrast, the PU fusion showed at least partial oncogenic activity. Furthermore, NIH-3T3 cells expressing the PU fusion grew significantly faster than the control cells over 72 h and showed an increase in the number of colony forming units compared with the vector control-expressing cells (**Figures 2E, F**).

Dicentric (7;9)(p11-p13;p11-p13) is a very rare but recurrent abnormality in BCP-ALL patients as well as a limited number of cases involving PAX5 rearrangement. Indeed, we identified only 7 cases of dic(7;9) from among approximately thousands of cases with karyotypic data (**Supplementary Table 1**). Most cases with the translocation, dicentric abnormality or derivatives of chromosomes 7 and 9 involving PAX5 rearrangement mainly presented PAX5-LOC392027, PAX5-POM121, PAX5-ELN, and PAX5-AUTS2 (4, 8–14). Some aberrant PAX5 transcripts have also been reported, such as a case of MPAL that harbored der(9)t(7;9)(q11.2;p13) (10). To our knowledge, this is the first case of

PAX5 rearrangement in a Ph-positive MAPL patient with dic(7;9). Previous studies showed that most malignant cells carrying PAX5 fusions displayed a simple karyotype (6). Coexistence of the t(9;22)(q34;q11) translocation, which resulted in the formation of the BCR-ABL1 p190 fusion in this study, might contribute to the cytogenetic complexity and suggest a poor prognosis. The partner genes involved in the PAX5 fusions were heterogeneous, but a partner involving a ubiquitin-related gene was the first to be reported. Previous reports indicated that half of the PAX5 fusion genes gave rise to truncated PAX5 proteins, including those involving out-of-frame fusions (6). Consistently, the PAX5-UBE2D4 fusion showed the competitive inhibition of wt-PAX5 transactivating activity, similar to truncated PAX5. Furthermore, the PAX5-UBE2D4 fusion presented oncogenic activity in a nude mouse model. In contrast, WT PAX5 showed tumor suppressive ability both *in vivo* and *in vitro*.

## Patient Perspective

Since the diagnosis, the patient received and understood the cause of his illness, and the possible cause of premature death. Ultimately, he hoped to get the right treatment.

## Data Availability Statement

The original contributions presented in the study are included in the article/**Supplementary Material**. Further inquiries can be directed to the corresponding authors.

## Ethics Statement

All procedures performed in studies involving human participants were in accordance with the ethical standards of the institutional and/or national research committee(s) and with the Helsinki Declaration (as revised in 2013). Written informed consent to participate in this study was provided by the participants’ legal guardian/next of kin.

## Author Contributions

YY, ZZ, JX, and QL contributed equally to this study and performed most of the experiments. HQ and SC were the principal investigators. AS, WC, RZ, JP, and YZ analyzed and discussed the data. All authors contributed to the article and approved the submitted version.

## Funding

This study was supported by grant from the National Key R&D Program of China (2019YFA0111000), the National Natural Science Foundation of China (81700140, 81873449, 81970142, 81900130, 81970136, 81970132), the Natural Science Foundation of the Jiangsu Higher Education Institution of China (18KJA320005, 18KJB320019), the Natural Science Foundation of Jiangsu Province (BK20190180), the priority academic program development of Jiangsu Higher Education Institution, the Innovation Capability Development Project of Jiangsu Province(BM2015004), the Translational Research Grant of NCRCH (2020WSB03, 2020WSB11, 2020WSB13) and the Open Project of Jiangsu Biobank of Clinical Resources (SBK202003001, SBK202003 003).

## Conflict of Interest

The authors declare that the research was conducted in the absence of any commercial or financial relationships that could be construed as a potential conflict of interest.

## Publisher’s Note

All claims expressed in this article are solely those of the authors and do not necessarily represent those of their affiliated organizations, or those of the publisher, the editors and the reviewers. Any product that may be evaluated in this article, or claim that may be made by its manufacturer, is not guaranteed or endorsed by the publisher.
