# Supplementary material for: Case Report: The Formation of a Truncated PAX5 Transcript in a Case of Ph-Positive Mixed Phenotype Acute Leukemia With dic(7;9)(p11-p13;p13)
Source: Front Oncol. 2021 Aug 26;11:703612. doi: 10.3389/fonc.2021.703612 (PMC8427297; doi:10.3389/fonc.2021.703612)

**Supplementary**

Supplementary Table 1. Baseline clinical characteristics of 7 patients with dic(7;9) chromosome abnormality screening from about 60000 patients in our medical center during the 27 years from 2007 to 2012 years.

| **Numbers** | **sex** | **age** | **Diagnosis** | **Cytogenetic abnormality** | **FISH (PAX5)** |
| --- | --- | --- | --- | --- | --- |
| P1 | M | 33 | CML | 45,XX,dic(7;9)(p11;p11)[11]/46,XX[1]. | N |
| P2 | M | 62 | CML-BC | 46,XY,t(9;22)(q34;q11)[6]45,idem,dic(7;9)[4]. | N |
| P3 | M | 33 | ALL | 45,XY,dic(7;9)(p11;p11)[11] | N |
| P4 | M | 45 | ALL | 46,XY,t(9;22)[6]/45,idem,dic(7;9)(p11;p13)[2] | N |
| P5 | M | 16 | MPAL | 45,XY,dic(7;9), t (9;22) [8]/46, XY [9] | PAX5-R |
| P6 | F | 16 | ALL | 45,XX,1q+?dup(1)(q32),dic(7;9)(p11;p11),inv(9)(p12q12),11q-[6]/46,XX, inv(9) (p12q12)[4] | N |
| P7 | F | 62 | CML-BC | 46,XX,dic(7;9)(p11;p11),t(9;22)(q34;q11),10q+[10]. | N |

**Abbreviations**: P1-7, patient 1-7; M, Male; F, Female; CML, Chronic Myelogenous Leukemia; BC, Blast Crisis; ALL, Acute Lymphoblastic Leukemia; MPAL, Mixed Phenotype Acute Leukemia. N, negative; PAX5-R, PAX5 rearrangement.

Supplementary Figure1: Flow cytometric analysis 23.4% of the BM blasts cells showed CD34，CD10，CD19，CD33，CD13，and CD79a positive.


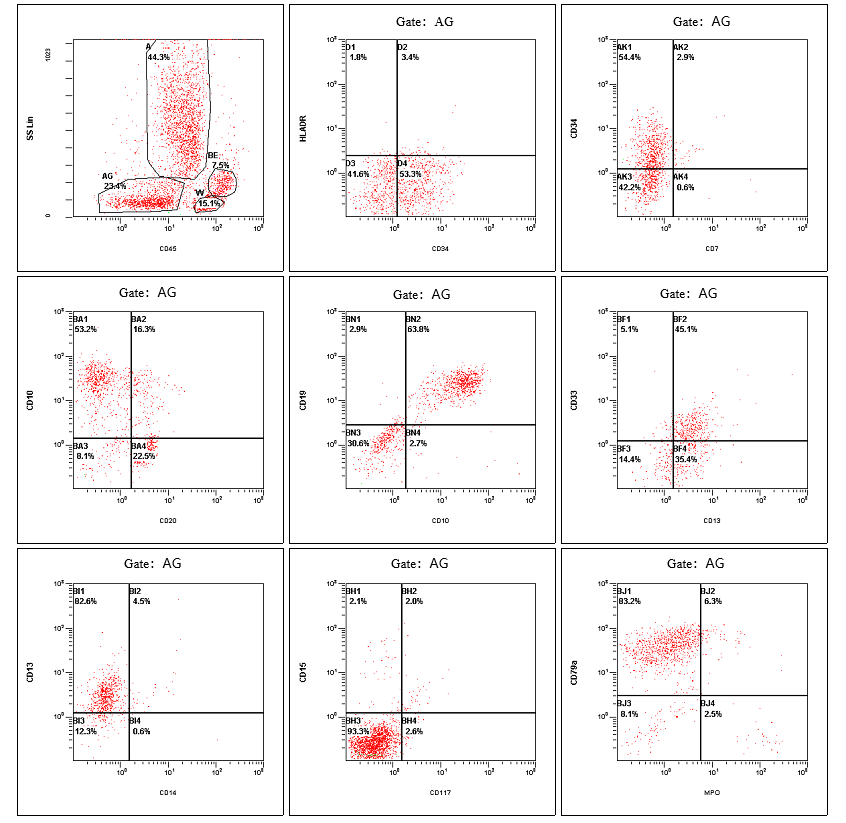


Supplementary Figure2. PAX5 probes for FISH: the BAC RP11-652D9 (red) and RP11-344B23 (green) correspond respectively to the 5’ part of the PAX5 gene up to intron 5 and the 3’ part of PAX5 from intron 6.

RP11-652D9

RP11-344B23


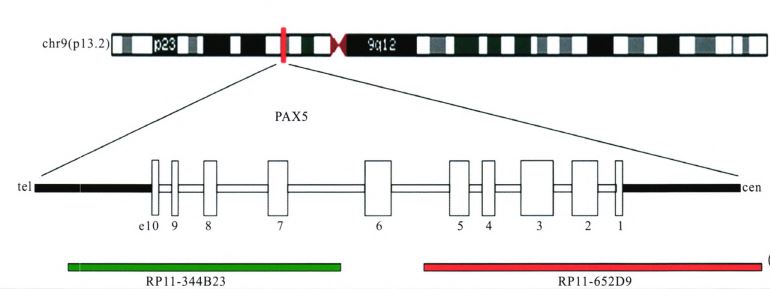


Supplementary Figure 3. PAX5-UBE2D4 (PU) fusion transcript was detected by reverse transcription polymerase chain reaction (RT-PCR) amplification with Forward primers: CTTGGCAGGTATTATGAGACA，and reverse primers: AACGGGTAATCTGTAGGAAAGT. Lane 2-6, indicate the patient 1 to 5 with dic(7;9); lane 7 for blank control. RNA samples of Patient 6 and 7 were not available.


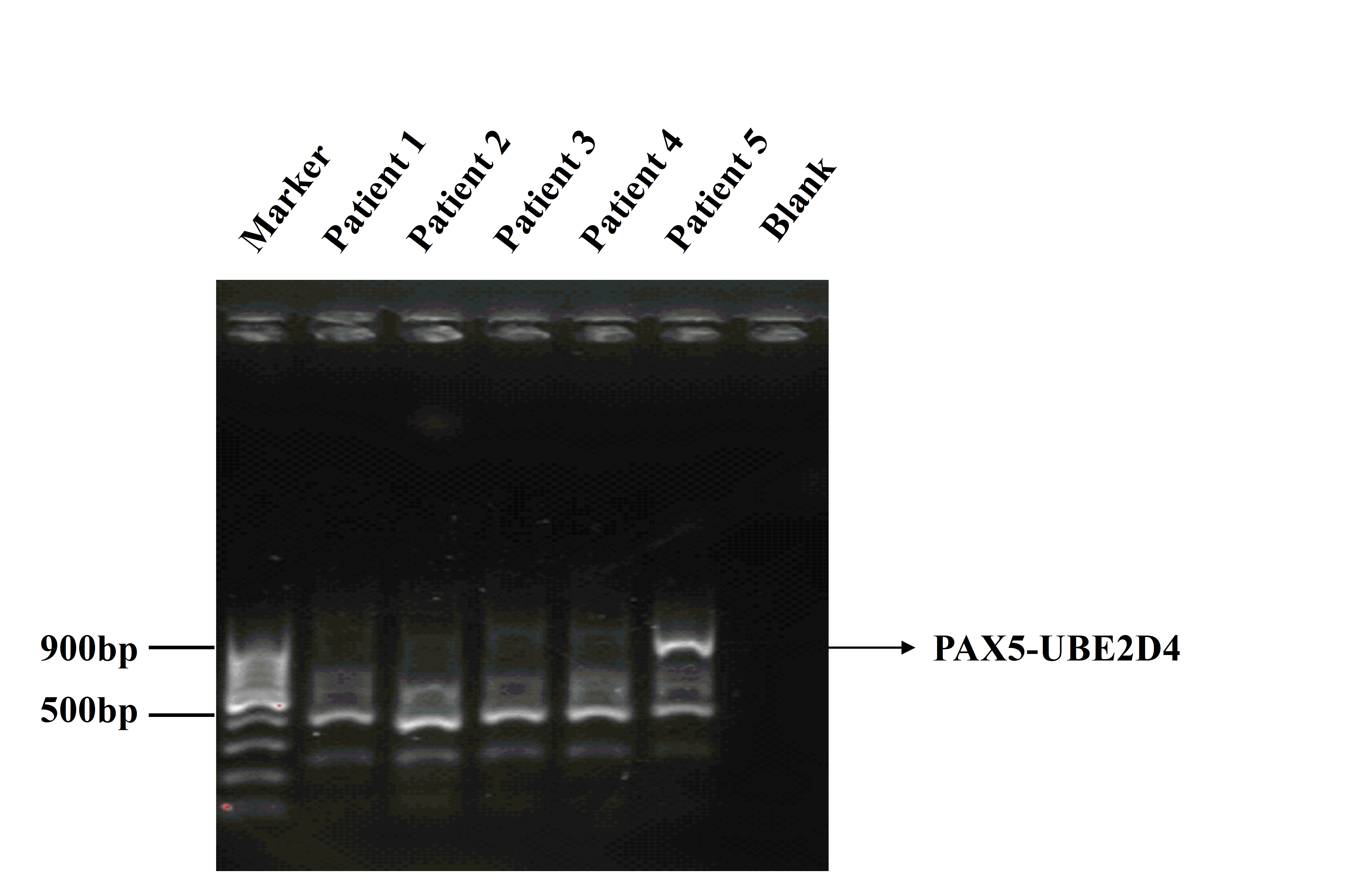


Supplementary Figure4. Immunofluorescence assay show the nucleus localization of PU fusion.


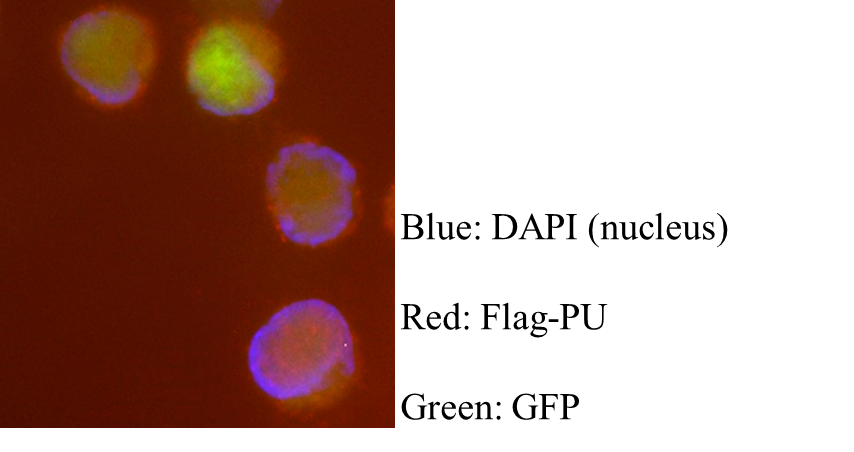


Supplementary Figure 5. The effect of the PAX5-UBE2D4 (PU) on the growth and the clonal formation of K562 cells with or without imatinib (IM).


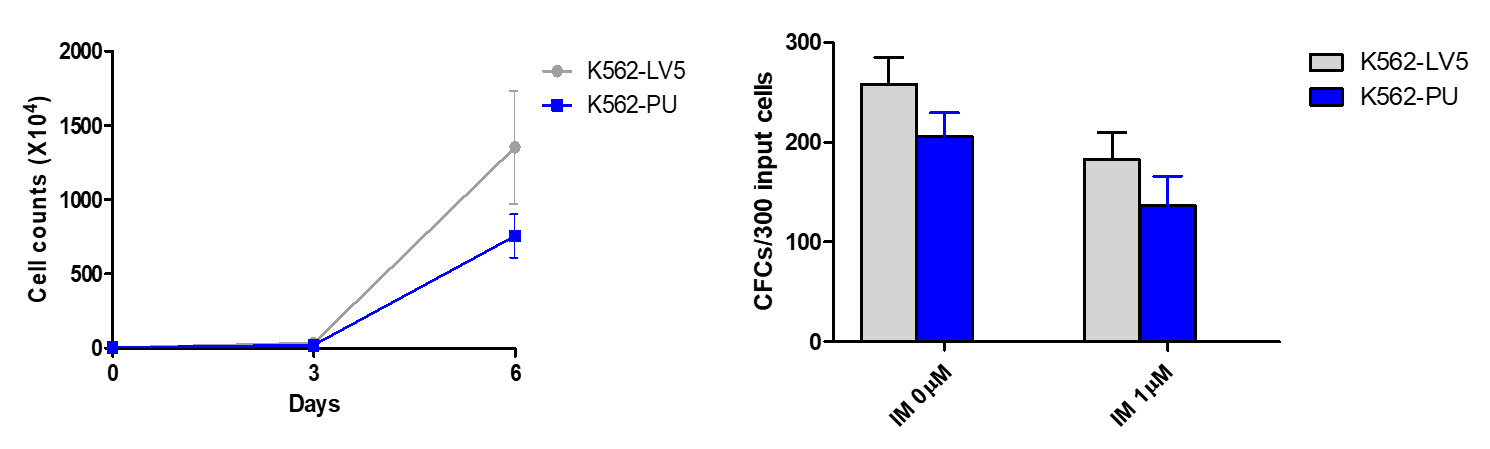


Supplementary Figure 6. The oncogenic activity of the PAX5-UBE2D4 (PU) fusion. (a) BaF3 cells were co-transfected with BCR-ABL (BA) and the vector (LV5) or PU. (b) Kaplan-Meier curves show the overall survival of BA-PU and BA-LV5 groups by intravenous injection of indicated BaF3 cells. (c)The weights of the liver and the spleen of mice with BaF3-BA-PU or BaF3-BA-LV5.


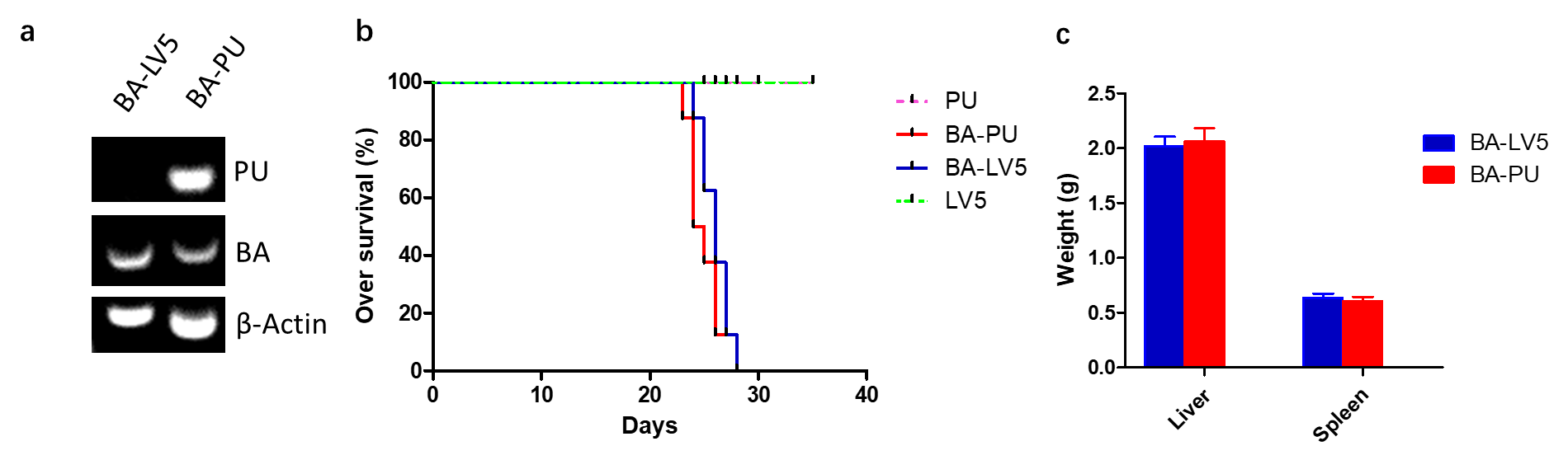


Supplementary Figure 7. The effect of the PAX5-UBE2D4 (PU) on the activity of ERK, stat5, and JNK signaling pathways in K562 cells.


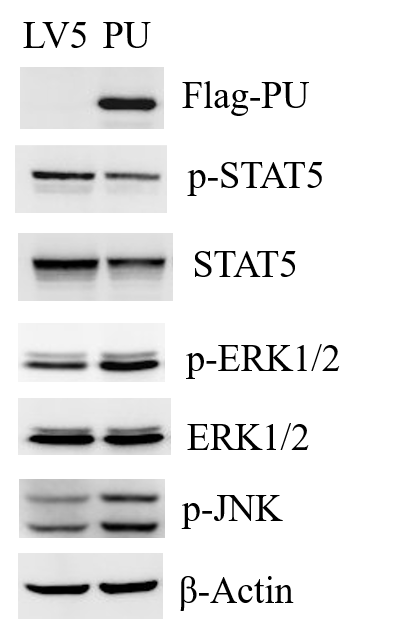

Supplement: Supplementary file 1 [file DataSheet_1.doc]
